# Stage IA Patients With Pancreatic Ductal Adenocarcinoma Cannot Benefit From Chemotherapy: A Propensity Score Matching Study

**DOI:** 10.3389/fonc.2020.01018

**Published:** 2020-07-16

**Authors:** Yuchao Zhang, Gang Xu, Maozhen Chen, Qian Wei, Tengteng Zhou, Ziliang Chen, Mingyang Shen, Ping Wang

**Affiliations:** ^1^Vascular Surgery, The Affiliated Huaian No. 1 People's Hospital of Nanjing Medical University, Huaian, China; ^2^Department of Breast Surgery, XuZhou Central Hospital, The Affiliated XuZhou Hospital of Medical College of Southeast University, Xuzhou, China; ^3^Department of Breast Surgery, Xuzhou Maternal and Child Health Hospital, Xuzhou, China

**Keywords:** pancreatic ductal adenocarcinoma, overall survival, chemotherapy, Surveillance, Epidemiology, End Results (SEER), prognosis

## Abstract

**Purpose:** Adjuvant chemotherapy following resection is recommended by clinical practice guidelines for all patients with pancreatic ductal adenocarcinoma (PDAC). This study aimed to evaluate the efficacy of adjuvant chemotherapy among the staging groups of the American Joint Committee on Cancer (AJCC) for PDAC.

**Patients and Methods:** This retrospective cohort analysis was performed by the Surveillance Epidemiology and End Results (SEER) (2004–2015) database and multi-institutional dataset (2010–2018). Baseline clinicopathologic characteristics of PDAC patients, including age, gender, ethnicity, marital status, education level, county income level, county unemployed rate, insurance status, grade, stage, chemotherapy, and radiotherapy, were collected. Overall survival (OS) was analyzed using the Kaplan–Meier method. The SEER and multi-institutional data were adjusted with 1:1 ratio propensity score matching (PSM).

**Results:** In total, 6,274 and 1,361 PDAC patients were included from the SEER database and multi-institutional dataset, respectively. Regardless of the count of resected lymph nodes, adjuvant chemotherapy prolonged the long-term OS time for stage IB, IIA, IIB, and III patients in both SEER and multi-institutional cohorts. Nevertheless, adjuvant chemotherapy did not provide additional clinical benefits even after a PSM adjustment for stage IA patients in both SEER and multi-institutional cohorts.

**Conclusion:** Adjuvant chemotherapy improved the long-term survival of stage IB, IIA, IIB, and III PDAC patients; however, it demonstrated no survival benefit in stage IA PDAC patients. Thus, adjuvant chemotherapy should not be recommended for stage IA PDAC patients. These would significantly reduce the economic burden of society and improve the life quality of stage IA PDAC patients.

## Introduction

Pancreatic ductal adenocarcinoma (PDAC) remains one of the most challenging malignancies to treat, even though surgical technique and systemic therapy have improved over the past decades. Due to concealed pathogenesis and rapid progress, only a small minority of PDAC patients undergo an operation. Consequently, PDAC has a lethality of more than 95% and poor prognosis in most cases (1, 2). Clinical treatment options vary according to the severity of PDAC. Curative resection is considered the only approach to cure resectable PDAC patients. The emergence of neoadjuvant therapy offers the potential for curative resection in borderline resectable patients with initially unresectable and locally advanced PDAC (3). Postoperative adjuvant chemotherapy is still an essential supplementation to further improve the prognosis of PDAC patients (4) and is recommended for all patients with PDAC following resection according to the European Society for Medical Oncology-European Society of Digestive Oncology (ESMO-ESDO) and National Comprehensive Cancer Network (NCCN) Clinical Practice Guidelines (5, 6).

Despite all attempts made to improve the survival rate of PDAC patients, a meta-analysis including five randomized controlled trials showed that adjuvant chemotherapy only provided an extra 3 months of median survival time for patients with resected PDAC (7). Considering that adjuvant chemotherapy may cause pain, nausea, tiredness, drowsiness, and breath shortness, clinicians should be cautious about the application of adjuvant chemotherapy. It has been reported that adjuvant chemotherapy has no favorable impact on the survival of early-stage patients in many malignancies such as ovarian cancer (8), lung cancer (9), gallbladder cancer (10), and colorectal cancer (11). In the current study, we performed a population-based and multi-institutional analysis on PDAC patients to evaluate the efficacy of adjuvant chemotherapy with an ultimate aim to investigate whether adjuvant chemotherapy was necessary for early-stage PDAC patients.

## Materials and Methods

### Ethics Statement

This study was approved by the institutional review board of The Affiliated Huaian No. 1 People's Hospital of Nanjing Medical University. Patients from the Surveillance, Epidemiology, and End Results (SEER) database had previously consented to participate in any scientific research worldwide.

### Patients

We selected patients with PDAC from the SEER database (2004–2015) and multi-institutional dataset (2010–2018). In the SEER database, all the cases were identified by the topographical code of “pancreas” (International Classification of Disease for Oncology, third edition, ICD-O-3) using SEER^*^Stat software (Version 8.2.0). The multi-institutional dataset was from The Affiliated Huaian No. 1 People's Hospital of Nanjing Medical University and Xuzhou Central Hospital, The Affiliated Xuzhou Hospital of Medical College of Southeast University. Inclusion criteria were as follows: (1) ≥18 years; (2) first primary PDAC with histological diagnosis; (3) without distant metastasis at diagnosis; (4) treatment with curative surgery; (5) definite number of resected lymph nodes; (6) definite staging groups according to the 8th Edition American Joint Committee on Cancer (AJCC) staging manual; and (7) definite information about radiotherapy and chemotherapy. Follow-up time ranged from 0 to 143 months in the SEER database and from 0 to 88 months in the multi-institutional dataset. Patients with unavailable follow-up information were excluded. The International Study Group on Pancreatic Surgery (ISGPS) recommended that at least 15 lymph nodes should be resected to assess the status of lymph nodes (12). Therefore, patients would be divided into two subgroups (15 or more lymph nodes evaluation, <15 lymph nodes evaluation) for further analysis. Baseline clinicopathologic characteristics of PDAC patients included age, gender, ethnicity, marital status, education level, county income level, county unemployed rate, insurance status, grade, stage, chemotherapy, and radiotherapy. Education level meant county education level in the SEER database. The variable “%< high school education (in tens) ACS 2011–2015” was used to evaluate the county education level in the SEER database, which was divided into quarters (low: <11.2, mid-low: 11.2–19.8, mid-high: 19.8–28.4, high: >28.4). Likewise, “median family income (in tens) ACS 2011–2015” variable was also divided into quarters (low: <5,300, mid-low: 5,300–7,700, mid-high: 7,700–10,150, high: >10,150), and “% unemployed ACS 2011–2015” variable was divided into quarters (low: <6.3, mid-low: 6.3–11.1, mid-high: 11.3–15.8, high: >15.8). Insurance status was classified as insured (including insured and any Medicaid), uninsured, and other (including unknown and blank). Notably, data for insurance status before 2007 were not available in the SEER database. In the multi-institutional dataset, education level meant individual education level and was divided into four levels: low (elementary school), mid-low (middle school), mid-high (university or college), and high (postgraduate).

### Statistical Analysis

All data were analyzed by IBM SPSS 22.0 software. The survival curves for overall survival (OS) were drawn using the Kaplan–Meier method. OS was defined as the interval from PDAC diagnosis until death or the last follow-up. The SEER and multi-institutional data were adjusted with 1:1 ratio propensity score matching (PSM). *P* < 0.05 was considered statistically significant.

## Results

In total, 6,274 PDAC patients were selected from the SEER database, including 503 at stage IA, 1,193 at stage IB, 449 at stage IIA, 2,555 at stage IIB, and 1,574 at stage III (Table 1). The median age was 66 years, and the majority was White (82.5%) and reported as insured (including Medicaid). Patients with middle levels, including income level, education level, and unemployed rate, made up the majority of the entire cohort. A total of 3,522 (56.1%) patients had well or moderately differentiated tumors (grade I + II), and 2,271 (36.2%) patients had poorly differentiated or undifferentiated tumors (grade III + IV). Of the entire cohort, less than half of the patients (39.3%) received radiotherapy. In addition, 4,353 (69.4%) patients received chemotherapy, while 1,921 (30.6%) patients did not.

**Table 1:** Baseline clinicopathologic characteristics of PDAC patients.

**Variables**	**SEER (*n* = 6,274)**	**Multi-institutional dataset (*n* = 1,361)**
**Age**		
Median (range)	66 (26–93)	57 (19–74)
**Gender**		
Male	3,194 (50.9%)	649 (47.7%)
Female	3,080 (49.1%)	712 (52.3%)
**Ethnicity**		
White	5,174 (82.5%)	0
Black	666 (10.6%)	0
Asian^a^	0	1,361 (100%)
Other	434 (6.9%)	0
**Marital status**		
Married	3,936 (62.7%)	1,187 (87.2%)
Other^b^	2,338 (37.3%)	174 (12.8%)
**Grade**		
I + II	3,522 (56.1%)	882 (64.8%)
III + IV	2,271 (36.2%)	422 (31.0%)
Unknown	481 (7.7%)	57 (4.2%)
**Stage**		
IA	503 (8.0%)	158 (11.6%)
IB	1,193 (19.0%)	299 (22.0%)
IIA	449 (7.2%)	207 (15.2%)
IIB	2,555 (40.7%)	473 (34.8%)
III	1,574 (25.1%)	224 (16.5%)
**Chemotherapy**		
Yes	4,353 (69.4%)	747 (54.9%)
No/unknown	1,921 (30.6%)	614 (45.1%)
**Radiotherapy**		
Yes	2,466 (39.3%)	306 (22.5%)
No	3,808 (60.7%)	1,055 (77.5%)
**County income level**		
Low	703 (11.2%)	-
Mid-low	3,310 (52.8%)	-
Mid-high	1,619 (25.8%)	-
High	642 (10.2%)	-
**Education level^c^**		
Low	178 (2.8%)	127(9.3%)
Mid-low	1,250 (19.9%)	478 (35.1%)
Mid-high	3,101 (49.5%)	673 (49.5%)
High	1,745 (27.8%)	83 (6.1%)
**Insurance status**		
Insured	4,778 (76.2%)	-
Uninsured	139 (2.2%)	-
Other^d^	1,357 (21.6%)	-
**County unemployed rate**		
Low	648 (10.3%)	-
Mid-low	4,425 (70.5%)	-
Mid-high	1,166 (18.6%)	-
High	35 (0.6%)	-

a *Born and grown up in Asia*.

b *Including single, divorced, and widowed, etc*.

c *County education level for the SEER database and individual education level for the multi-institutional data*.

d *Including unknown and blank*.

*PDAC, pancreatic ductal adenocarcinoma; SEER, Surveillance, Epidemiology, and End Results*.

**Figure 1:**
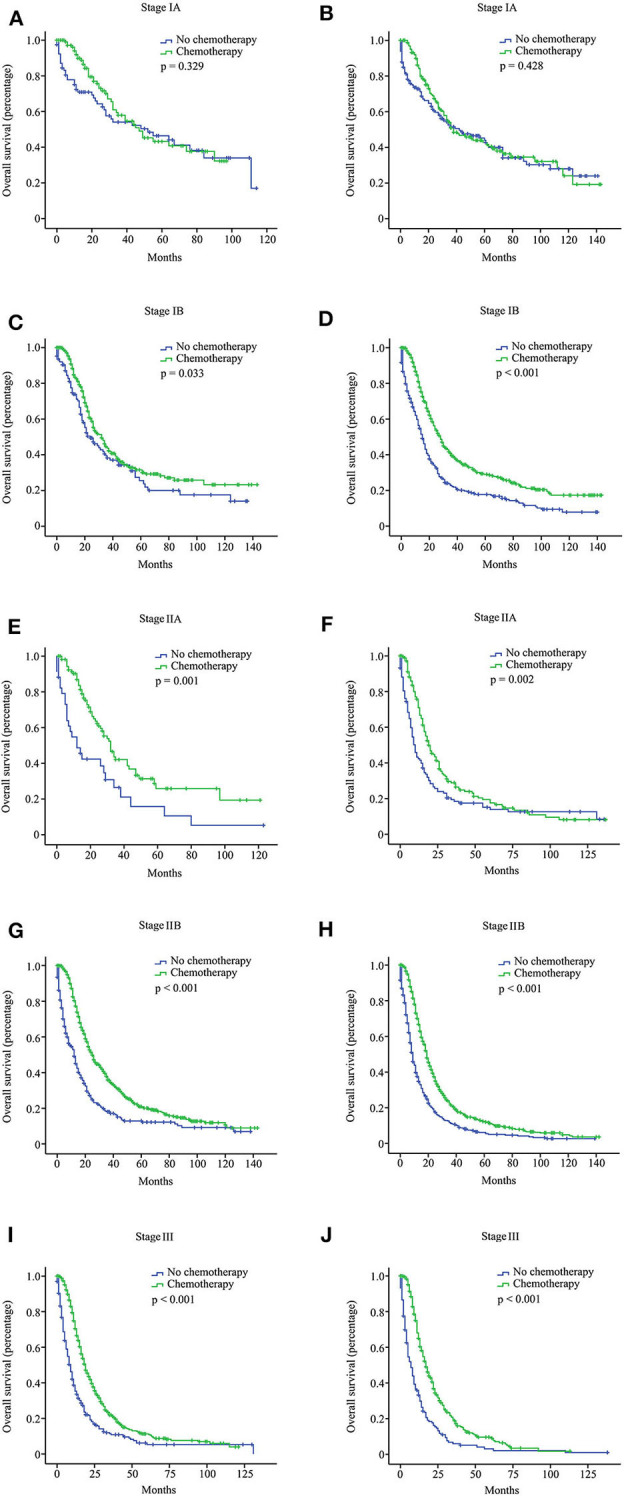
Overall survival (OS) curves for pancreatic ductal adenocarcinoma (PDAC) patients with different stages from the Surveillance, Epidemiology, and End Results (SEER) database according to the 8th American Joint Committee on Cancer (AJCC) staging system. Stage IA with 15 or more resected lymph nodes **(A)**; stage IA with <15 resected lymph nodes **(B)**; stage IB with 15 or more resected lymph nodes **(C)**; stage IB with <15 resected lymph nodes **(D)**; stage IIA with 15 or more resected lymph nodes **(E)**; stage IIA with <15 resected lymph nodes **(F)**; stage IIB with 15 or more resected lymph nodes **(G)**; stage IIB with <15 resected lymph nodes **(H)**; stage III with 15 or more resected lymph nodes **(I)**; stage III with <15 resected lymph nodes **(J)**.

**Figure 2:**
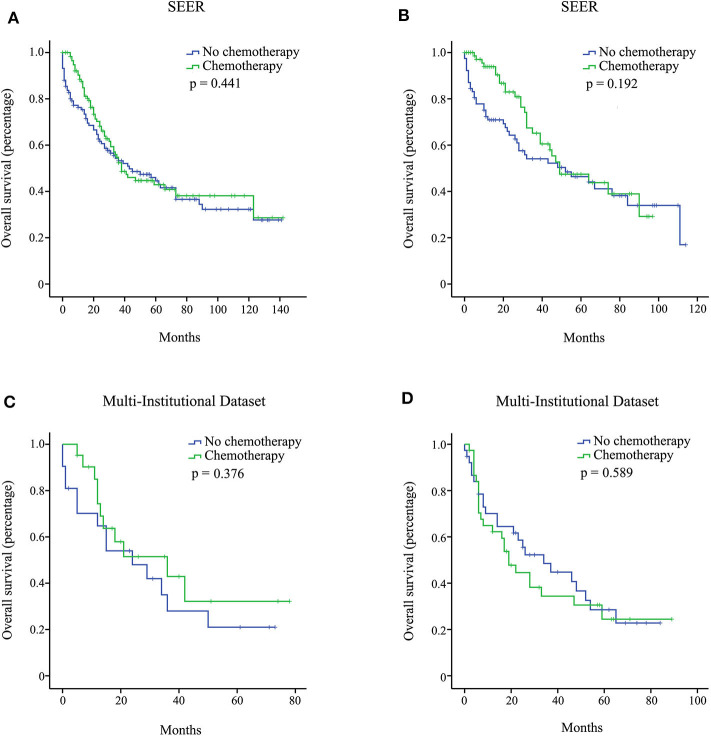
Overall survival (OS) curves for stage IA pancreatic ductal adenocarcinoma (PDAC) patients from the Surveillance, Epidemiology, and End Results (SEER), and multi-institutional dataset after propensity score matching (PSM) adjustment. Stage IA with 15 or more resected lymph nodes from the SEER database **(A)**; stage IA with <15 resected lymph nodes from the SEER database **(B)**; stage IA with 15 or more resected lymph nodes from the multi-institutional dataset **(C)**; stage IA with <15 resected lymph nodes from the multi-institutional dataset **(D)**.

We investigated the effect of chemotherapy on patients at each staging group from the SEER database (Figure 1). Regardless of the count of resected lymph nodes, chemotherapy prolonged the long-term OS time for stage IB, IIA, IIB, and III patients but not for stage IA patients. After PSM adjustment for clinically relevant covariates (including age, gender, grade, ethnicity, radiotherapy, and marital status), 117 pairs of stage IA patients with 15 or more resected lymph nodes and 78 pairs of stage IA patients with <15 resected lymph nodes were included in further analysis, respectively. As a result, there was still no survival difference between patients with chemotherapy and those without chemotherapy regardless of the count of resected lymph nodes (*p* > 0.05; Figures 2A,B). Additionally, we provided the cancer-specific survival (CSS) plots in Supplementary Figure 1. Similar results were observed. In particular, there was almost a statistically significant survival difference between patients with chemotherapy and those without chemotherapy for stage IB with 15 or more resected lymph nodes (*p* = 0.054).

**Figure 3:**
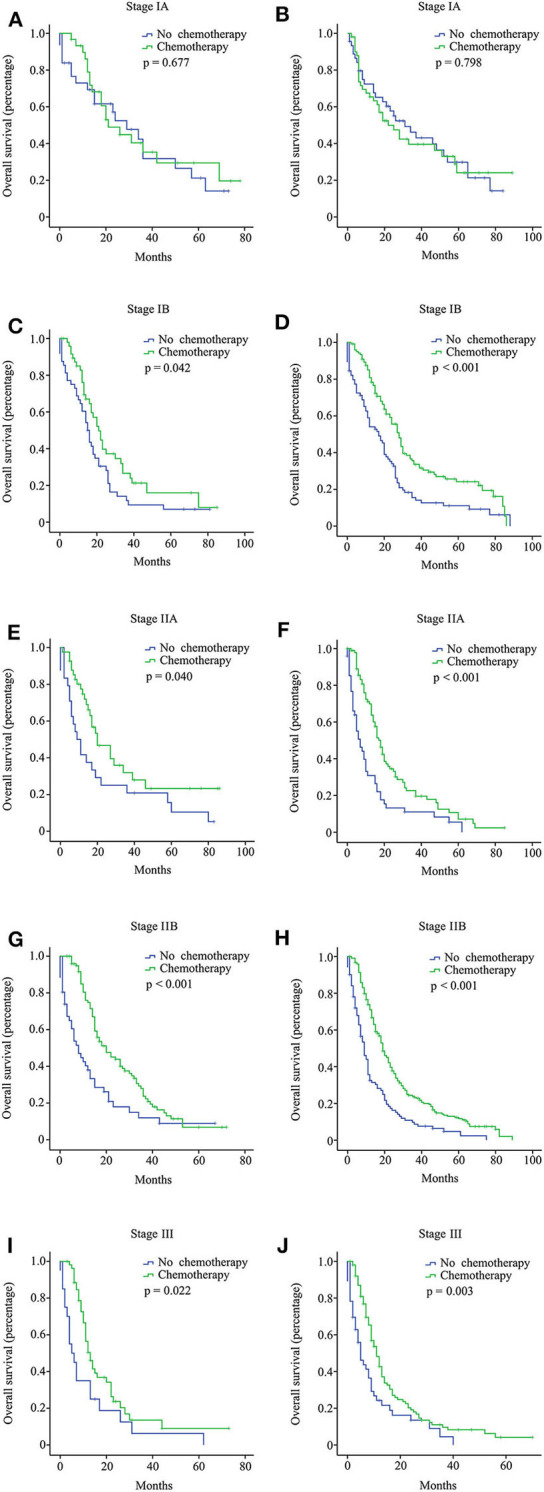
Overall survival (OS) curves for pancreatic ductal adenocarcinoma (PDAC) patients with different stages from the multi-institutional dataset according to the 8th American Joint Committee on Cancer (AJCC) staging system. Stage IA with 15 or more resected lymph nodes **(A)**; stage IA with <15 resected lymph nodes **(B)**; stage IB with 15 or more resected lymph nodes **(C)**; stage IB with <15 resected lymph nodes **(D)**; stage IIA with 15 or more resected lymph nodes **(E)**; stage IIA with <15 resected lymph nodes **(F)**; stage IIB with 15 or more resected lymph nodes **(G)**; stage IIB with <15 resected lymph nodes **(H)**; stage III with 15 or more resected lymph nodes **(I)**; stage III with <15 resected lymph nodes **(J)**.

In the multi-institutional dataset (Table 1), 1,361 PDAC patients met the inclusion criterion, including 158 cases at stage IA, 299 cases at stage IB, 207 cases at stage IIA, 473 cases at stage IIB, and 224 cases at stage III. The median age was 57 years, and all patients were Asian. A total of 882 (64.8%) patients had tumors at grade I + II, and 422 (31.0%) patients had tumors at grade III + IV. Among the patients, 77.5% did not receive radiotherapy. In addition, 747 (54.9%) patients received chemotherapy, while 614 (45.1%) patients did not. Similarly, the survival analysis showed that chemotherapy prolonged the long-term OS time for stage IB, IIA, IIB, and III patients but not for stage IA patients (Figure 3). After PSM adjustment, similar results were observed that chemotherapy did not provide clinical benefits for stage IA patients (Figures 2C,D).

## Discussion

In this study, we analyzed the SEER and multi-institutional dataset to evaluate the influence of adjuvant chemotherapy on survival in PDAC patients with different staging groups and found that adjuvant chemotherapy demonstrated no survival benefit on stage IA PDAC patients but was conducive to improve the survival rate of patients with other stages (stages IB, IIA, IIB, and III). The result provided new evidence for individualized treatment and questioned the current recommendation in the ESMO-ESDO and NCCN clinical practice guidelines for early-stage PDAC patients. These would significantly reduce the economic burden of society and improve the life quality of patients.

Adjuvant chemotherapy provided survival benefits for PDAC patients indeed (13–15), which our study also supported. However, adjuvant chemotherapy seemed irrelevant to long-term survival for stage IA PDAC patients based on our analysis. Most studies reported resectable PDAC patients as a single unit for investigating the roles of adjuvant chemotherapy, including ESPAC-1, ESPAC-3, ESPAC-4, CONKO-001, and JASPAC-01 (4, 16–23). Few studies focused primarily on the early-stage PDAC patients. Hamura et al. (24) classified 81 cases of stage I PDAC patients into invasive subgroup and non-invasive subgroup according to whether there was tumor invasion around the pancreas. The study indicated that adjuvant chemotherapy may improve OS for the invasive subgroup but not for the non-invasive subgroup. According to the 7th edition AJCC staging manual, Ostapoff et al. (25) showed that adjuvant chemotherapy was associated with better OS outcomes for stage I PDAC (including stage IA and IB) using the National Cancer Data Base (NCDB). Also using the NCDB, however, Shaib et al. (26) further reported that adjuvant chemotherapy did not improve the prognosis for stage I sub-centimeter PDAC (<1 cm in greatest dimension). Although the classification methods in our study varied from the previous studies, these results indicated that early-stage PDAC patients may not benefit from adjuvant chemotherapy.

The difference in sensitivity to adjuvant chemotherapy between stage IA PDAC patients and PDAC patients with more advanced stages is likely rooted in genetic alterations. PDAC mainly arises from non-invasive pancreatic intraepithelial neoplasms (27), whose histologic progression (from hyperplasia, atypia, carcinoma *in situ* to invasive ductal adenocarcinoma) is highly correlated with the accumulation of genetic alterations (28). For instance, oncogenic *KRAS* mutation itself generates the earliest pancreatic hyperplasia (29), and its combination with inactivated *TP53* and *SMAD4* induces invasive carcinomas (29). Chromatin-remodeling complex *SWI/SNF* has also been revealed to drive the development of PDAC significantly (30). More epigenetic and genetic drivers of PDAC are being identified. However, it is still a riddle how the order of these mutations or abnormalities influence clinical presentation and disease outcome of PDAC. In 2015, Ortmann et al. (31) reported that the order in which *JAK2* and *TET2* mutations were acquired in patients with myeloproliferative neoplasms influenced clinical features and the response to targeted therapy, which give us a hint that the sensitivity of PDAC at different stages to adjuvant chemotherapy may stem from the difference of key drivers and mutation order, which shape certain characteristics of early-stage and advanced PDAC.

A more backhanded reason may be the distinction of inner microenvironment of PDAC at different stages. As an inflammatory malignance, PDAC has exclusive pathological characteristics, with abundant cellular components, including cancer cells, pancreatic stellate cells (PSCs), cancer-associated fibroblasts, and tumor-associated macrophages, etc. (32). Varieties of cellular and molecular mechanisms are involved in tumor progression and resistance to chemotherapy. As PDAC progresses, both the proportion of each kind of cells and the extracellular matrix change. As opposed to PDAC patients at advanced stages which have complex components, such as the promotion of the angiogenesis, lymphangiogenesis, and induction of immunosuppressive reactions (33), early-stage PDAC patients mainly comprises of cancer cells and PSCs (34, 35). Upon adjuvant chemotherapy, the tumor microenvironment gets remodeled as each kind of cell reacts to the drugs (36–39). The difference in sensitivity to adjuvant chemotherapy between stage IA PDAC patients and PDAC patients with more advanced stages may be relevant to the complexity of tumor microenvironment and the various reactions of cells to chemotherapeutic drugs.

There are a few limitations in our study. First, the SEER database did not provide the data about recurrence, and the actual efficacy of the adjuvant chemotherapy could not be estimated fully. Second, the data of SEER and multi-institutional dataset were retrospective. More prospective analysis is necessary to validate the current conclusion. Third, detailed chemotherapy regimens were not recorded in the SEER database. Currently, most of the adjuvant chemotherapy regimens are based on gemcitabine (40) or fluorouracil (41). Other drugs such as oxaliplatin (42) and irinotecan (43) may be more suited to palliative treatment. In the study, all the chemotherapy regimens were regarded as a single unit, and it cannot be excluded whether a particular drug may play a favorable role in the prognosis of stage IA PDAC patients.

In sum, our analysis showed that current adjuvant chemotherapy demonstrated no survival benefit on stage IA PDAC patients, and their clinical treatment should be reevaluated accordingly.

## Data Availability Statement

Publicly available datasets were analyzed in this study. This data can be found here: https://seer.cancer.gov/data/.

## Ethics Statement

This study was approved by the institutional review board of The Affiliated Huaian NO. 1 People's Hospital of Nanjing Medical University. Patients from the Surveillance, Epidemiology, and End Results (SEER) database had previously consented to participate in any scientific research worldwide.

## Author Contributions

YZ and PW made substantial contributions to the design of the study, carried out the analysis, and interpreted the data. GX and MC contributed to the review of previous literature. ZC and MS contributed substantially to the data discussion and critically commented on the manuscript for scientific content. All authors made substantial contributions to data interpretation and drafting of the manuscript and were responsible for the quality of the overall manuscript, and approved the final version of the manuscript.

## Conflict of Interest

The authors declare that the research was conducted in the absence of any commercial or financial relationships that could be construed as a potential conflict of interest.

## Supplementary Material

Supplementary Figure 1 CSS curves for PDAC patients with different stages from the SEER database according to the 8th AJCC staging system. Stage IA with 15 or more resected lymph nodes **(A)**; stage IA with <15 resected lymph nodes **(B)**; stage IB with 15 or more resected lymph nodes **(C)**; stage IB with <15 resected lymph nodes **(D)**; stage IIA with 15 or more resected lymph nodes **(E)**; stage IIA with <15 resected lymph nodes **(F)**; stage IIB with 15 or more resected lymph nodes **(G)**; stage IIB with <15 resected lymph nodes **(H)**; stage III with 15 or more resected lymph nodes **(I)**; stage III with <15 resected lymph nodes **(J)**.
